# Three new species of woodlizards (Hoplocercinae, *Enyalioides*) from northwestern South America

**DOI:** 10.3897/zookeys.494.8903

**Published:** 2015-04-06

**Authors:** Omar Torres-Carvajal, Pablo J. Venegas, Kevin de Queiroz

**Affiliations:** 1 Museo de Zoología, Escuela de Ciencias Biológicas, Pontificia Universidad Católica del Ecuador, Avenida 12 de Octubre y Roca, Apartado 17-01-2184, Quito-Ecuador; 2 Department of Vertebrate Zoology, National Museum of Natural History, Smithsonian Institution, MRC 162, Washington, DC 20560, USA; 3 División de Herpetología-Centro de Ornitología y Biodiversidad (CORBIDI), Santa Rita N˚105 Of. 202, Urb. Huertos de San Antonio, Surco, Lima-Perú

**Keywords:** Andes, Ecuador, *Enyalioides*, Hoplocercinae, Iguania, lizards, new species, Peru, systematics

## Abstract

The discovery of three new species of *Enyalioides* from the tropical Andes in Ecuador and northern Peru is reported. *Enyalioides altotambo* **sp. n.** occurs in northwestern Ecuador and differs from other species of *Enyalioides* in having dorsal scales that are both smooth and homogeneous in size, a brown iris, and in lacking enlarged, circular and keeled scales on the flanks. *Enyalioides anisolepis* **sp. n.** occurs on the Amazonian slopes of the Andes in southern Ecuador and northern Peru and can be distinguished from other species of *Enyalioides* by its scattered, projecting large scales on the dorsum, flanks, and hind limbs, as well as a well-developed vertebral crest, with the vertebrals on the neck at least three times higher than those between the hind limbs. *Enyalioides sophiarothschildae* **sp. n.** is from the Amazonian slopes of the Cordillera Central in northeastern Peru; it differs from other species of *Enyalioides* in having caudal scales that are relatively homogeneous in size on each caudal segment, a white gular region with a black medial patch and several turquoise scales in males, as well as immaculate white labials and chin. A molecular phylogenetic tree of 18 species of hoplocercines is presented, including the three species described in this paper and *Enyalioides cofanorum*, as well as an updated identification key for species of Hoplocercinae.

## Abstract (es)

Reportamos el descubrimiento de tres especies nuevas de *Enyalioides* de los Andes tropicales en Ecuador y norte de Perú. *Enyalioides altotambo* **sp. n.**, del noroccidente de Ecuador, difiere de otras especies de *Enyalioides* por poseer escamas dorsales lisas y homogéneas en tamaño, iris café y por carecer de escamas circulares grandes y quilladas en los flancos. *Enyalioides anisolepis* **sp. n.** ocurre en las estribaciones amazónicas de los Andes al sur de Ecuador y norte de Perú, y se distingue de otras especies de *Enyalioides* por poseer escamas grandes y proyectadas dispersas en el dorso, flancos y extremidades posteriores, así como por su cresta vertebral bastante desarrollada, que a nivel del cuello es tres veces más alta que entre las extremidades posteriores. *Enyalioides sophiarothschildae* **sp. n.**, de las estribaciones amazónicas de la Cordillera Central al norte del Perú, difiere de otras especies de *Enyalioides* por poseer escamas caudales de tamaño similar en cada segmento caudal, una región gular blanca con una mancha medial negra y escamas turquesa en machos, así como la quijada y labiales de color blanco. También presentamos un árbol filogenético molecular de 18 especies de hoplocercinos, que incluye a las tres especies descritas en este artículo y a *Enyalioides cofanorum*, así como una clave de identificación actualizada para las especies de Hoplocercinae.

## Introduction

The iguanian lizard clade *Hoplocercinae* includes 16 currently recognized species assigned to *Enyalioides*, *Hoplocercus*, and *Morunasaurus* distributed from Panama to central Brazil (Torres-Carvajal et al. 2011). Woodlizards (*Enyalioides*) occupy lowland tropical rainforests including the Chocó and the western Amazon basin, with nine species (75%) occuring east of the Andes and three (25%) occuring west of the Andes.

With nearly 40% of the total number of species described in the last seven years from Ecuador and Peru (Torres-Carvajal et al. 2008; Torres-Carvajal et al. 2009; Venegas et al. 2011; Venegas et al. 2013), woodlizards represent one of the South American lizard groups with the highest species discovery rate (corrected for clade size) in the last decade. This is a striking fact given that woodlizards are among the largest and most colorful lizards in South American tropical forests and is most likely the result of recent fieldwork in poorly explored areas of the central and northern Andes. Here we contribute to this growing body of taxonomic knowledge with the description of three new species of *Enyalioides*, one from the Pacific slopes of the Andes in northern Ecuador, and the other two from the Amazonian slopes of the Andes in southern Ecuador and northern Peru.

## Materials and methods

Snout-vent length (SVL) and tail length (TL) measurements were made with a ruler and recorded to the nearest millimeter. All other measurements (i.e., head width, length and height; rostral and mental width and height) were made with digital calipers and recorded to the nearest 0.1 mm. Sex was determined by noting the presence of hemipenes or sexually dichromatic characters. The format of Torres-Carvajal et al. (2011) is followed for the descriptions of the new species, as well as the terminology of these authors for scutellational characters and measurements. Specimens of other species of *Enyalioides* examined in this study are listed in the Appendix. The distribution map was constructed in ArcMap 9.3 (ESRI, Inc.); WGS84 is the datum for all coordinates presented below. Institutional abbreviations correspond to Museo de Zoología, Pontificia Universidad Católica del Ecuador (QCAZ), Quito; Centro de Ornitología y Biodiversidad (CORBIDI), Lima, Peru; Museo de Historia Natural San Marcos (MUSM), Lima, Peru.

### Phylogenetic analyses

Following laboratory protocols similar to those presented by Torres-Carvajal and de Queiroz (2009), we sequenced a continuous 1773 base fragment of mitochondrial DNA (mtDNA) that extends from the gene encoding subunit I of the protein NADH dehydrogenase (ND1) through the genes encoding tRNA^Ile^, tRNA^Gln^, tRNA^Met^, subunit II of NADH dehydrogenase (ND2), tRNA^Trp^, tRNA^Ala^, tRNA^Asn^, the origin of light-strand replication (OL), tRNA^Cys^, tRNA^Tyr^, to the gene encoding subunit I of the protein cytochrome c oxidase (COI). We added five new sequences from the new species described herein and one of *Enyalioides cofanorum* (QCAZ 8035) to the mtDNA dataset of Venegas et al. (2013). GenBank accession numbers for the new sequences are provided in Table 1.

**Table 1.** Vouchers, locality data, and GenBank accession numbers of new DNA sequences obtained for this study.

Taxon	Voucher	Locality	GenBank number (ND4)	GenSeq nomenclature
*Enyalioides altotambo*	QCAZ 8073 (holotype)	Ecuador: Esmeraldas: Alto Tambo, 5 km on road to Placer	KP235211	genseq-1
*Enyalioides anisolepis*	QCAZ 8395	Ecuador: Zamora-Chinchipe: Chito, sector Los Planes	KP235213	genseq-2
*Enyalioides anisolepis*	QCAZ 8428	Ecuador: Zamora-Chinchipe: Chito	KP235214	genseq-2
*Enyalioides anisolepis*	QCAZ 8515	Ecuador: Zamora-Chinchipe: Chito, sector Los Planes	KP235215	genseq-2
*Enyalioides cofanorum*	QCAZ 8035	Ecuador: Orellana: 66 km on road Pompeya-Iro	KP235210	genseq-4
*Enyalioides sophiarothschildae*	CORBIDI 647 (holotype)	Peru: San Martín: Río Lejía on the trail La Cueva-Añazco Pueblo	KP235212	genseq-1

Editing, assembly, and alignment of sequences were performed with Geneious 7.1.7 (Drummond et al. 2010). Genes were combined into a single dataset with four partitions, three corresponding to each codon position in protein coding genes and one to all tRNAs. The best partition strategy along with the corresponding models of evolution were obtained in PartitionFinder 1.1.1 (Lanfear et al. 2012) under the Bayesian information criterion.

Phylogenetic relationships were assessed under a Bayesian inference approach using MrBayes 3.2.2 (Ronquist et al. 2012) after partitioning the data as described above. To reduce the chance of converging on a local optimum, four runs were performed. Each consisted of five million generations and four Markov chains with default heating values. Trees were sampled every 1000 generations resulting in 5001 saved trees per analysis. Stationarity was confirmed by plotting the –ln *L* per generation in the program Tracer 1.6 (Rambaut et al. 2013). Additionally, the standard deviation of the partition frequencies and the potential scale reduction factor (Gelman and Rubin 1992) were used as convergence diagnostics for the posterior probabilities of bipartitions and branch lengths, respectively. Adequacy of mixing was assessed by examining the effective sample sizes (ESS) in Tracer, with ESS > 200 considered as satisfactory. After analyzing convergence, mixing, and sampling, the first 501 trees in the sample were discarded as “burn-in” from each run. We then confirmed that the four analyses had reached stationarity at a similar likelihood score and that the topologies were similar and used the resultant 18,000 trees to calculate posterior probabilities (PP) for each bipartition on a 50% majority rule consensus tree.

## Results

The taxonomic conclusions of this study are based on the observation of morphological features and color patterns, as well as the inferred phylogenetic relationships. This information is considered as species delimitation criteria following a general lineage or unified species concept (de Queiroz 1998; 2007).

### Enyalioides altotambo sp. n.

Taxon classificationAnimaliaSquamataHoplocercidae

http://zoobank.org/4AE55600-2B8F-446B-B702-B55BDC3FF1EF

Enyalioides altotambo Proposed standard English name: Alto Tambo woodlizards
Enyalioides altotambo Proposed standard Spanish name: lagartijas de palo de Alto Tambo
Enyalioides oshaughnessyi (part) Torres-Carvajal et al. 2011: 23.

#### Type material.

**Figure 1.**
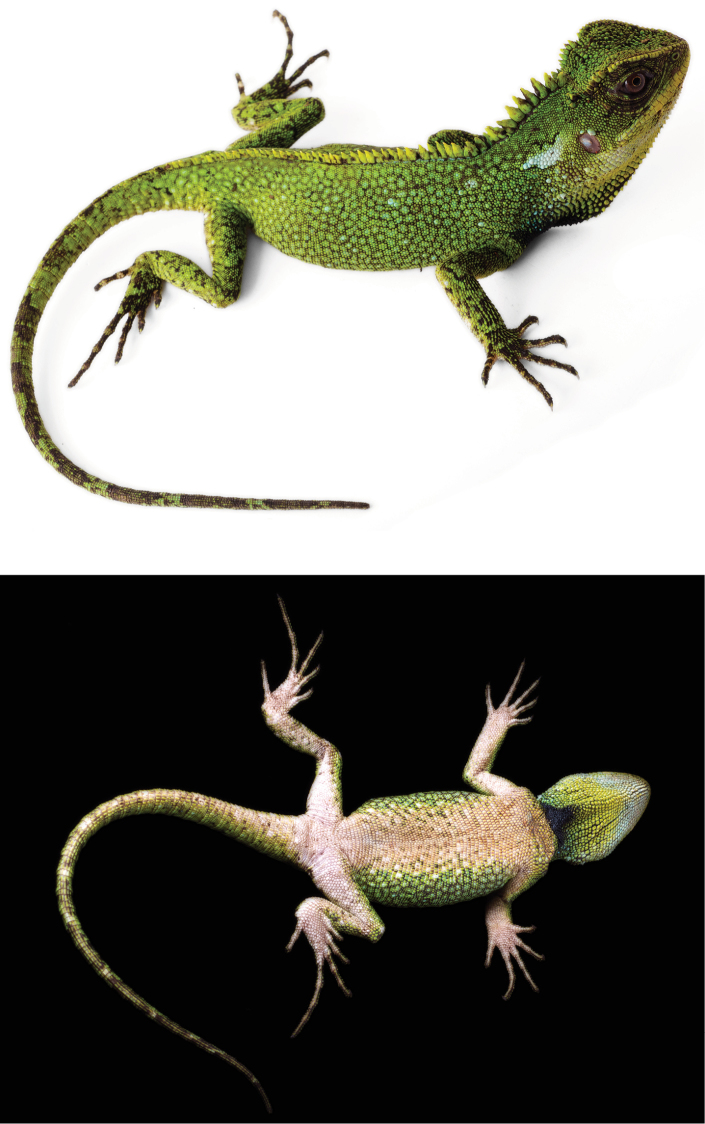
Holotype (QCAZ 8073, adult male, SVL = 119 mm) of *Enyalioides altotambo* in dorsal (top) and ventral (bottom) views. Photographs by Luis A. Coloma.

*Holotype*. QCAZ 8073 (Fig. 1), an adult male from Alto Tambo, 5 km on road to Placer, Bosque Integral Otokiki, 0.90600°N; -78.60600°W (DD), 620 m, Provincia Esmeraldas, Ecuador, collected on 2 May 2010 by I.G. Tapia, D. Almeida-Reinoso, J.M. Guayasamin and L.A. Coloma.

*Paratype*. ECUADOR: Provincia Esmeraldas: QCAZ 6671, adult female, Alto Tambo, Balthazar river, 0.90000°N; -78.61667°W, 645 m, collected on 5 November 2005 by F. Ayala-Varela and I.G. Tapia.

#### Diagnosis.

*Enyalioides altotambo* differs from other species of *Enyalioides*, except for *Enyalioides oshaughnessyi*, in having dorsal scales that are both smooth and homogeneous in size. It can be distinguished from *Enyalioides oshaughnessyi* (character states in parentheses) by the following characters: iris brown in both sexes (iris bright red in both sexes); scales on lateral edge of skull roof just posterior to superciliaries strongly projected (moderately projected); adults of both sexes with light green spots on dorsum (if present, spots turquoise or blue); adult males with a black medial patch on gular region not extending dorsally to form an antehumeral bar (black patch under gular fold extending dorsally to form a short antehumeral bar); scales on flanks almost homogenous in size (flank scales heterogeneous in size, with a few enlarged, circular, keeled scales); pale postympanic stripe on lateral aspect of neck in both sexes (pale postympanic spot in both sexes), posterior surface of thighs without enlarged scales (scattered enlarged scales), tail length/total length 0.57–0.60 (0.59–0.62).

#### Description of holotype.

Male (Fig. 1); SVL = 119 mm; TL = 160 mm; maximum head width = 21.9 mm; head length = 29.8 mm; head height = 20.3 mm; dorsal head scales keeled or multicarinate, projected dorsally; parietal eye present; eight scales immediately posterior to superciliaries conical, dorsolaterally projected, and conspicuously larger than adjacent scales; temporal scales small, pyramidal, low; one large conical pretympanic scale; superciliaries 17; canthals five; postrostrals three; supralabials 13 if counted to a point below middle of eye; rostral divided into three small scales, similar in size to adjacent supralabials; one longitudinal row of lorilabials between suboculars and supralabials at level of middle of eye, longitudinal rows of lorilabials anterior to this point two; loreal region with small, keeled, and juxtaposed scales; nasal at level of supralabials V–VI; infralabials 11 if counted to a point below middle of eye; mental (1.68 mm wide × 1.98 mm high) slightly wider and 1.5 times higher than adjacent infralabials; postmentals three; gulars ventrally projected and separated from each other by skin covered with tiny granular scales; gular fold complete midventrally, extending dorsally and posteriorly to form an antehumeral fold; neck with some oblique folds, and a dorsolateral row of enlarged scales; distal aspect of oblique fold immediately anterior to antehumeral fold with approximately six enlarged scales similar in size to gulars, but more than three or four times the size of adjacent fold scales.

Vertebral crest strongly projected and decreasing in size posteriorly, with vertebrals on neck at least four times higher than those between hind limbs; crest bifurcates at a point approximately 10 mm posterior to the cloaca, and extends onto tail about 1/3 its length; body flanks between fore and hind limbs with slight dorsolateral fold; scales on dorsolateral fold slightly larger than adjacent scales; dorsal and flank scales small, smooth, imbricate, more or less homogeneous in size; ventral scales imbricate, keeled, rectangular or rhomboid, with a posterolateral mucron; ventrals more than twice the size (area) of dorsals.

Limb scales keeled dorsally and keeled or feebly keeled ventrally; scales on dorsal and posterior aspects of thighs heterogeneous in size, with most scales less than half the size of those scales on anterior and ventral aspects, separated from each other by skin covered with tiny granular scales; subdigitals on finger IV 25; subdigitals on toe IV 29; femoral pores on each side one; tail laterally compressed and gradually tapering posteriorly; caudal scales smooth at the base of tail, becoming keeled and imbricate towards tip, gradually increasing in size posteriorly on lateral and dorsal aspects of each caudal segment; caudals larger ventrally than dorsally; individual caudal segments three scales long ventrally and seven scales long dorsally.

#### Color in life of holotype

(Fig. 1). Head light green with a few black and dark brown scales; superciliaries, canthals and labials yellow; bluish cream blotch, wider than high, behind tympanum; pretympanic scales bluish cream; dorsal body background light green with a fine dark brown reticulation and scattered bluish cream scales; vertebrals yellowish green; tail green with incomplete dark brown rings; black irregular marks on limbs, covering most of hands and feet; chin white; gular region bluish cream anterolaterally grading into yellowish green and then bluish green posteriorly, with a posteromedial black patch; ventral aspect of body, limbs and tail dirty cream; flank color pattern extending onto ventrolateral aspect of body; iris brown with golden ring around pupil.

#### Variation.

**Figure 2.**
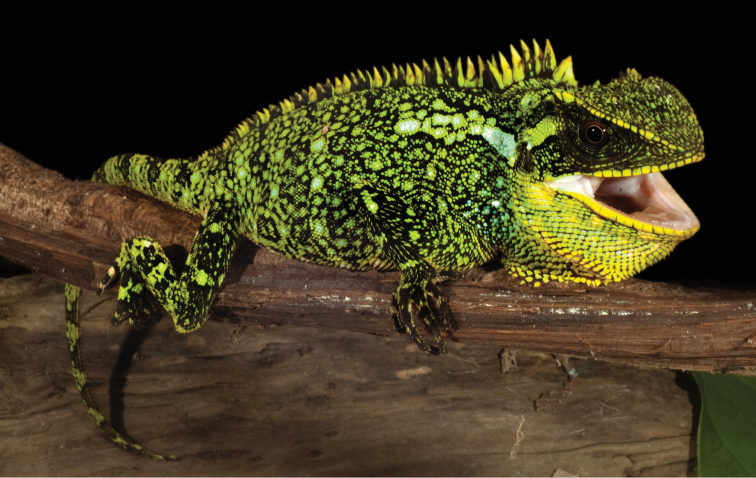
Paratype (QCAZ 6671, adult female, SVL = 132 mm) of *Enyalioides altotambo*. Photograph by Luis A. Coloma.

Variation in meristic and morphometric characters of *Enyalioides altotambo* are presented in Table 2. The single female paratype (QCAZ 6671; Fig. 2) is similar in lepidosis and color patterns to the holotype. It differs from the holotype in lacking a black gular patch, and in having a longer pale postympanic stripe, a yellow chin, and a yellow gular region. Furthermore, the scales on the lateral edge of the skull roof and those forming the vertebral crest are more projected in the female (Fig. 2); however, this variation could be ontogenetic rather than sexual because the female is larger (SVL = 134 mm) than the male (SVL = 119).

**Table 2.** Summary of morphological characters and measurements (mm) of *Enyalioides altotambo*, *Enyalioides anisolepis* and *Enyalioides sophiarothschildae*. Range (first line) and mean ± standard deviation (second line) are given for quantitative characters, except when there was no variation.

Character	*Enyalioides altotambo* *N* = 2	*Enyalioides anisolepis* *N* = 15	*Enyalioides sophiarothschildae* *N* = 3
Dorsals in transverse row between dorsolateral crests at midbody	39–40 39.5 ± 0.71	28–35 32.00 ± 2.83	22–26 24.33 ± 2.08
Ventrals in transverse row at midbody	31–33 32.00 ± 1.41	23–29 26.53 ± 1.92	23–26 25.00 ± 1.73
Vertebrals from occiput to base of tail	50–51 50.50 ± 0.71	43–62 50.87 ± 6.27	51–57 54.00 ± 3.00
Gulars	47	30–35 31.71 ± 1.49	36
Infralabials	11	9	9–11 10.00 ± 1.00
Supralabials	13	10–12 10.77 ± 0.60	9–12 10.67 ± 1.53
Canthals	5	5–6 5.43 ± 0.51	5
Superciliaries	14–17 15.50 ± 2.12	13–18 15.57 ± 1.40	13–15 14.00 ± 1.00
Transverse rows of ventrals between fore and hind limbs	47–49 48.00 ± 1.41	38–46 41.27 ± 2.60	37–40 38.67 ± 1.53
Subdigitals finger IV	23–25 24.00 ± 1.41	15–20 18.36 ± 1.39	18–19 18.67 ± 0.58
Subdigitals toe IV	27–29 28.00 ± 1.41	24–27 25.14 ± 0.86	22–27 25.33 ± 2.89
Femoral pores	1–2 1.50 ± 0.71	0–3 1.64 ± 1.01	3–4 3.67 ± 0.58
Tail length/Total length	0.57–0.60 0.59 ± 0.02	0.59–0.71 0.62 ± 0.03	0.60–0.61 0.61 ± 0.01

#### Distribution.

**Figure 3.**
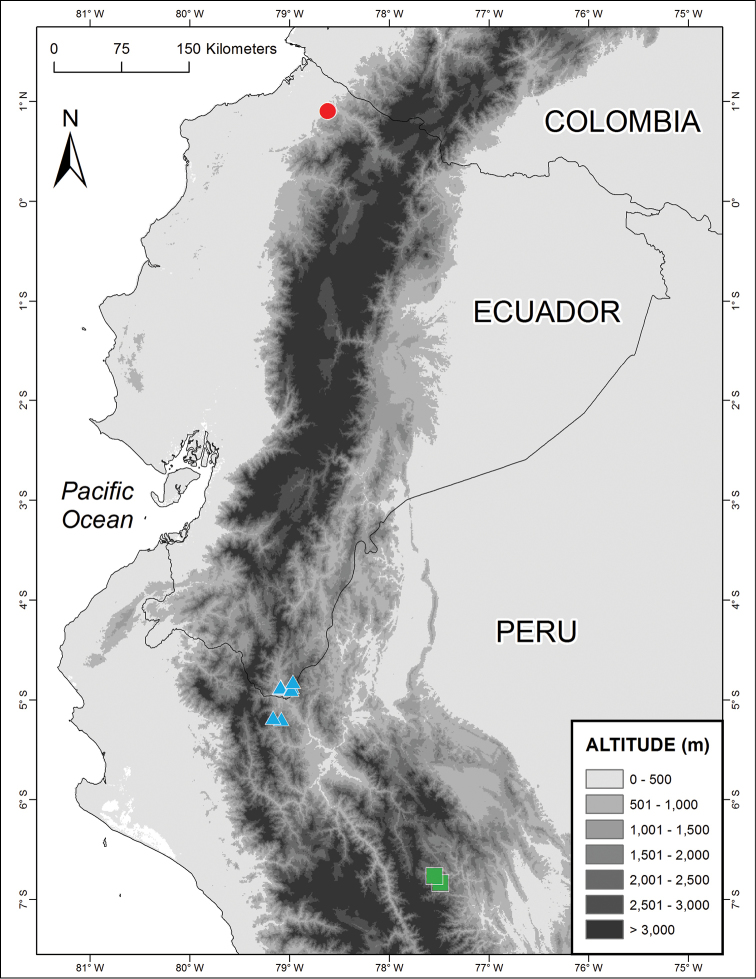
Distribution of *Enyalioides altotambo* (circles), *Enyalioides anisolepis* (triangles) and *Enyalioides sophiarothschildae* (squares).

*Enyalioides altotambo* is only known from two adjacent localities at 620–645 m in the Chocoan rainforests of northwestern Ecuador (Fig. 3). Female paratype QCAZ 6671 was found at 5:30 pm with its head facing up on a tree trunk.

#### Etymology.

The specific epithet is a noun in apposition and refers to Alto Tambo, Provincia Esmeraldas, Ecuador, a village on the Ibarra-San Lorenzo road where *Enyalioides altotambo* was discovered.

#### Remarks.

**Figure 4.**
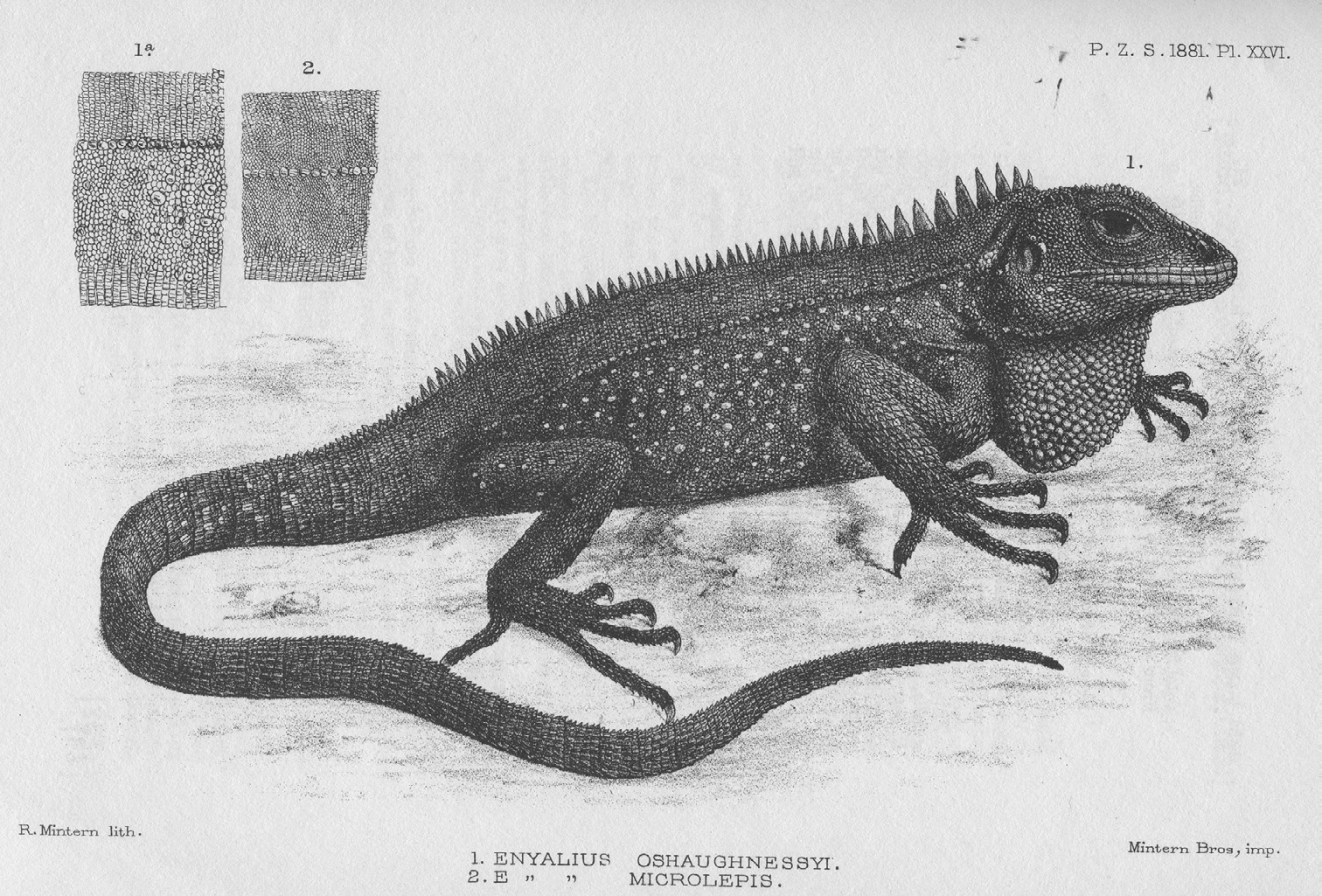
Holotype of *Enyalioides oshaughnessyi* (MRHN [Museum Royal d’Histoire Naturelle, Belgium] 2009, adult male). Illustration taken from original description (Boulenger 1881).

Although previously referred to *Enyalioides oshaughnessyi*, the possibility that the specimens here named *Enyalioides altotambo* represented a distinct species was recognized in previous studies. In a phylogenetic analysis of hoplocercine lizards, Torres-Carvajal and de Queiroz (2009) found “*Enyalioides oshaughnessyi*” to be paraphyletic relative to *Enyalioides touzeti* based on three samples of “*Enyalioides oshaughnessyi*”. One of them corresponded to the paratype of *Enyalioides altotambo*, and was sister to a clade containing the sister taxa *Enyalioides touzeti* and *Enyalioides oshaughnessyi*. Torres-Carvajal et al. (2011) noted that the color of the iris in live specimens of “*Enyalioides oshaughnessyi*” from Alto Tambo was not bright red as in live specimens of “*Enyalioides oshaughnessyi*” from other localities and suggested that the two forms represented separate species. Nonetheless, these authors found no other differences between the two potential species and refrained from associating the name *Enyalioides oshaughnessyi* with one versus the other because the type locality data of *Enyalioides oshaughnessyi* is vague (“Ecuador”), and the color of the iris was not recorded in its original description (Boulenger 1881). Here we recognize known populations other than that at Alto Tambo as *Enyalioides oshaughnessyi* based on the enlarged, circular and keeled scales scattered on the flanks of *Enyalioides oshaughnessyi* (absent in *Enyalioides altotambo*), as described and illustrated in its original description (Fig. 4; Boulenger 1881).

### Enyalioides anisolepis sp. n.

Taxon classificationAnimaliaSquamataHoplocercidae

http://zoobank.org/6728260C-76AD-4C46-B97B-158C44BDA70C

Enyalioides anisolepis Proposed standard English name: rough-scaled woodlizards
Enyalioides anisolepis Proposed standard Spanish name: lagartijas de palo de escamas ásperas

#### Type material.

**Figure 5.**
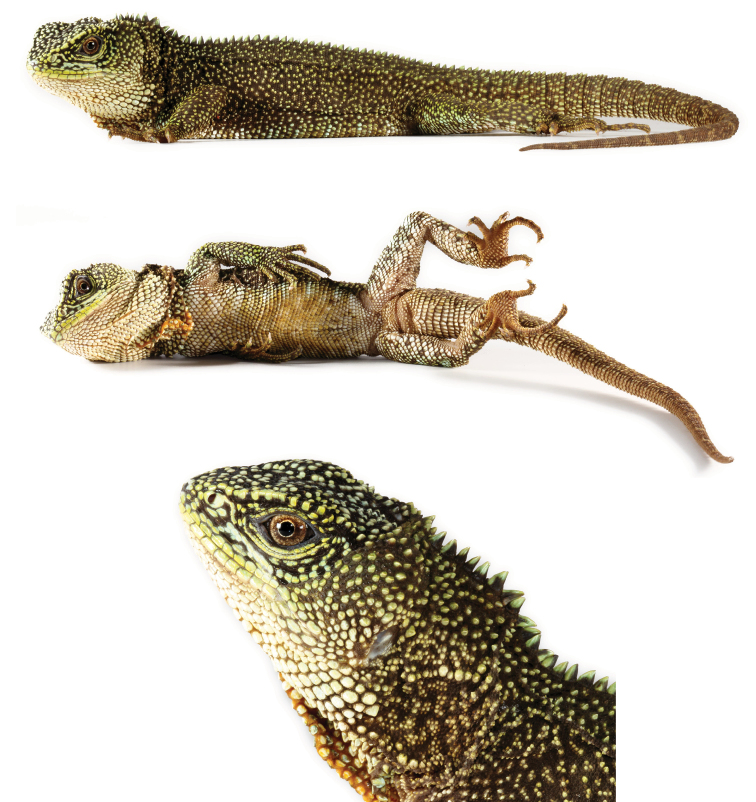
Holotype of *Enyalioides anisolepis* (QCAZ 12537, adult male, SVL = 130 mm). Top: dorsolateral view; middle: ventral view; bottom: lateral view of head. Photographs by Omar Torres-Carvajal.

*Holotype*. QCAZ 12537 (Fig. 5), an adult male from the eastern bank of the Mayo river, 4.5 km ESE Zumba, -4.88605°S, -79.08738°W (DD), 765 m, Provincia Zamora-Chinchipe, Ecuador, collected on 11 April 2014 by D.A. Paucar, D. Almeida-Reinoso, G. Galarza and D. Pareja.

**Figure 6.**
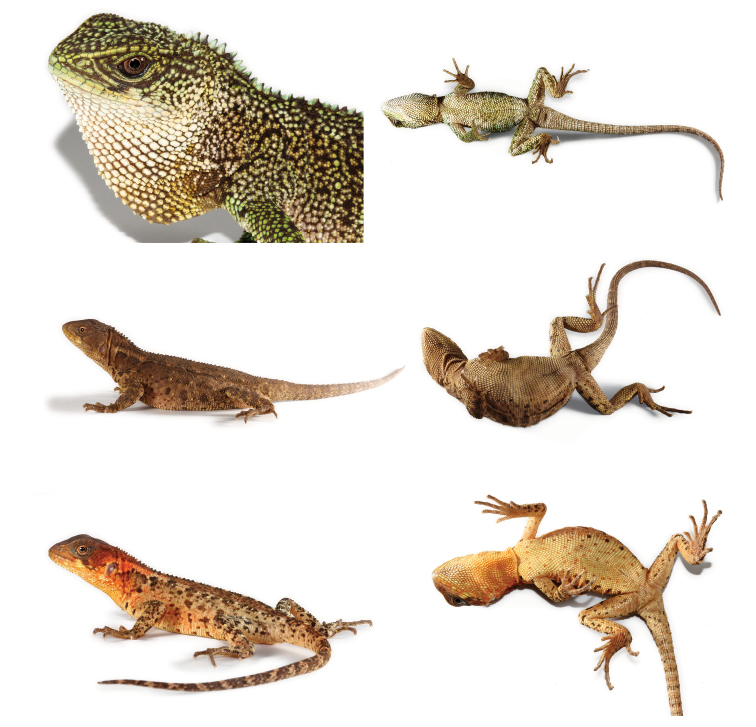
Paratypes of *Enyalioides anisolepis*. Lateral (left) and ventral (right) views of an adult male (top, QCAZ 12527, SVL = 111 mm), an adult female (middle, QCAZ 12552, SVL = 101 mm), and a juvenile (bottom, QCAZ 12535, SVL = 59 mm). Photographs by Omar Torres-Carvajal.

*Paratypes (14)*. ECUADOR: Provincia Zamora-Chinchipe: QCAZ 12521, juvenile with the same collection data as the holotype except -4.88673°S, -79.08744°W, 738 m; QCAZ 12527, adult male (Fig. 6) with the same collection data as the holotype except -4.87147°S, -79.08542°W, 738 m; QCAZ 12528, juvenile with the same collection data as the holotype except -4.87136°S, -79.08534°W, 738 m; QCAZ 12531, female with the same collection data as the holotype except -4.87808°S, -79.08956°W, 738 m; QCAZ 12535, juvenile (Fig. 6) with the same collection data as the holotype except -4.88658°S, -79.08747°W, 731 m; QCAZ 12536, juvenile with the same collection data as the holotype except -4.88622°S, -79.08737°W, 744 m; QCAZ 12552, female (Fig. 6) with the same collection data as the holotype except -4.87589°S, -79.08995°W, 741 m; QCAZ 12551, juvenile with the same collection data as the holotype except -4.87521°S, -79.08965°W, 724 m, collected on 12 April 2014; QCAZ 12517, adult male from Nuevo Paraíso, 700 m NW on road to Las Tres Aguas, -4,87109°S, -78,97579°W, 1742 m, collected on 10 April 2014 by the same collectors as the holotype; QCAZ 8395, female from Chito, sector Los Planes, -4.89814°S, -78.98095°W, collected on 16 February 2008 by S. Aldás-Alarcón; QCAZ 8515, female from Chito, sector Los Planes, -4.89726°S, -78.98191°W, collected on 18 February 2008 by S. Aldás-Alarcón; QCAZ 8428, female from Chito, 4.82037°S, -78.96247°W, 1724 m, collected on 14 February 2008 by S. Aldás-Alarcón. PERU: Provincia San Ignacio: Región Cajamarca: CORBIDI 870, female from Alto Ihuamaca-Namballe, -5.19448°S, -79.08048°W, 1616 m, collected on 26 August 2008 by M. Dobiey; MUSM 20675, adult female from El Sauce, Tabaconas Namballe National Sanctuary, -5.17897°S, -79.16347°W, 1600 m, collected in April 2003 by C. Aguilar.

#### Diagnosis.

**Figure 7.**
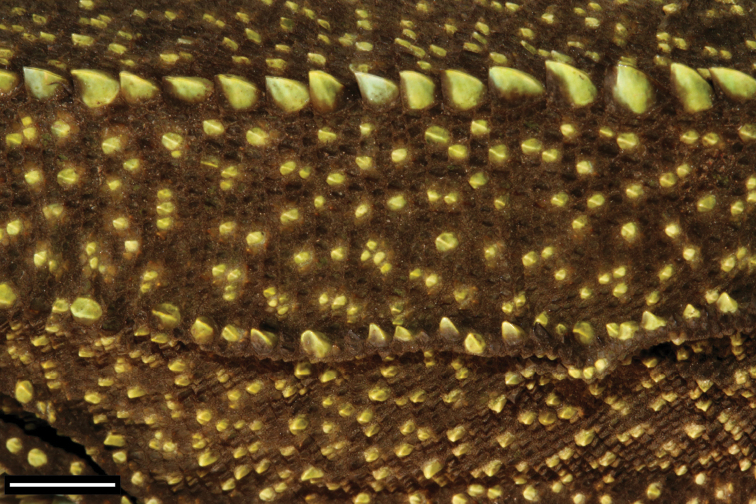
Close-up of left dorsum of *Enyalioides anisolepis* (QCAZ 12537, holotype) showing scattered enlarged scales. Scale bar = 5 mm. Photograph by Omar Torres-Carvajal.

*Enyalioides anisolepis* can be distinguished from other species of *Enyalioides*, except for *Enyalioides heterolepis*, by having conical dorsal head scales (only in *Enyalioides anisolepis* and *Enyalioides heterolepis*) and scattered, projecting, large scales on the dorsum, flanks, and hind limbs (also in *Enyalioides/Morunasaurus annularis* and *Enyalioides/Morunasaurus groi*), which are conspicuous in adults of both sexes (Fig. 7). Besides occurring on opposite sides of the Andes, *Enyalioides anisolepis* differs from *Enyalioides heterolepis* (character states from Torres-Carvajal et al. 2011 in parentheses) in having fewer vertebral scales, 43–62, 50.87 ± 6.27 (52–98, 74.61 ± 10.39), a higher vertebral crest with the vertebrals on neck at least three times higher than those between the hind limbs (vertebrals on neck maximum twice as high as those between hind limbs), scattered dark spots on belly in juveniles and adults of both sexes (belly without scattered dark spots, blackish medially in some adult males), tail in adult males moderately compressed laterally (strongly compressed), and a marked sexual dichromatism (Fig. 6), with the dorsal background color greenish in males and brownish in females (both sexes with a brownish background).

The only other species of *Enyalioides* with scattered, projecting dorsal scales is *Enyalioides cofanorum*, which differs from *Enyalioides anisolepis* in lacking projecting scales on the hind limbs, and in being smaller in size (maximum SVL in males and females of *Enyalioides cofanorum* 107 mm and 109 mm, respectively; 130 mm and 119 mm in *Enyalioides anisolepis*). Additionally, adults of both sexes of *Enyalioides cofanorum* have a brownish background (marked sexual dichromatism in *Enyalioides anisolepis*).

#### Description of holotype.

Male (Fig. 5); SVL = 130 mm; TL = 220 mm; maximum head width = 28.7 mm; head length = 35.3 mm; head height = 24.6 mm; dorsal head scales keeled or multicarinate, those in the parietal region strongly projected dorsally; scales immediately posterior to superciliaries conical and dorsally projected, forming longitudinal row of ten (left) or nine (right) scales that extends posteriorly over supratemporal region; temporal scales small, tuberculate or keeled, juxtaposed; one enlarged pretympanic scale; superciliaries 17; canthals six; postrostrals three; supralabials 11 if counted to a point right below middle of eye, and 16 if counted to commisure of mouth; rostral (2.6 mm wide × 1.5 mm high) about twice as wide as adjacent supralabials; single longitudinal row of lorilabials between suboculars and supralabials at level of middle of eye, longitudinal rows of lorilabials anterior to this point 2–4; loreal region with small, smooth and keeled, juxtaposed scales; nasal at level of supralabials III and IV; infralabials nine (left) or eight (right) if counted to a point right below middle of eye, and 13 (left) and 11 (right) if counted to commisure of mouth; mental (2.8 mm wide × 1.5 mm high) wider and higher than adjacent infralabials; postmentals two; gulars projected, low; gular fold complete midventrally; small dewlap present; neck with several longitudinal and oblique folds.

Vertebral crest strongly projected and decreasing in size posteriorly, with vertebrals on neck at least three times higher than those between hind limbs; crest bifurcates at a point approximately 10 mm posterior to the cloaca, and extends onto tail about ¼ its length; flanks between fore and hind limbs with dorsolateral and ventrolateral longitudinal folds, as well as several oblique folds; axillary region with three vertical folds; scales on dorsolateral folds slightly larger than adjacent scales giving the fold the appearance of a low crest; scales between dorsolateral folds and vertebral crest heterogeneous in size, prominently keeled, and imbricate, with largest scales twice as large as smallest ones; neck and scapular region with scattered, large conical scales; flank scales ventral to dorsolateral folds similar to those dorsal to folds, with largest scales four times as large as smallest ones (Fig. 7); axillary region with conical scales forming two short vertical folds; ventral scales imbricate, keeled, rhomboidal, with a posterior mucron; ventrals more than three times the area of smallest dorsals.

Limb scales keeled dorsally and ventrally, homogeneous in size on fore limbs; scales on dorsal and posterior aspect of thighs heterogeneous in size, with most scales less than half the size of those on anterior and ventral aspects; scales on dorsal surface of shanks heterogeneous in size, with granular scales between large keeled scales; subdigitals on finger IV 17; subdigitals on toe IV 25; three femoral pores on left leg, two on right leg; tail laterally compressed and gradually tapering posteriorly; caudal scales strongly keeled and imbricate, increasing in size posteriorly on lateral and dorsal aspects of each caudal segment; caudals larger ventrally than dorsally; individual caudal segments three scales long ventrally and six scales long dorsally.

#### Color in life of holotype

(Fig. 5). Dorsal and lateral aspects of head with scattered black, brown, and pale green scales; labials greenish cream; dorsal background of body, limbs and tail brownish green with scattered pale green scales; vertebral crest pale green, the base and posterior surface of each vertebral scale dark brown; gular scales cream, the skin between them gray; orange patch on medial aspect of throat; chest and belly cream with a pale orange tint; ventral surface of limbs dirty cream with scattered brown spots; ventral surface of tail dirty cream proximally and brown distally; iris pale brown peripherally with dark brown reticulations, dark brown centrally; thin golden ring around pupil.

#### Variation.

Variation in meristic and morphometric characters of *Enyalioides anisolepis* are presented in Table 2. Enlarged pretympanic scales are absent in more than half of the specimens; when present, they vary between 1–3. A few specimens have smooth scales intermixed with the keeled dorsals. Ventrals are keeled except for an adult female specimen (QCAZ 8428) that has smooth ventrals, and an adult male specimen (QCAZ 12517) that has smooth ventrals anteriorly and feebly keeled ventrals posteriorly. Caudal segments are 6–8 scales long laterally.

This species has a marked sexual dichromatism in background colors (green in males, brown in females; see Fig. 6). Adult male paratypes are very similar in color patterns to the holotype, except for having dark spots on the belly. A subadult male (QCAZ 12517) has scattered black flecks on the gular region.

Adult females share similar color patterns with juveniles (Fig. 6): dorsal background of head, body, limbs and tail dark or pale brown; flanks dark or pale brown with scattered dark spots, blotches, or transverse bands (cream flecks in QCAZ 8428); diagonal subocular dark stripe extending from subocular region to commisure of mouth; faint cream stripe extending longitudinally from tympanum to scapular region, except in specimen QCAZ 8428, which has instead a cream blotch posterior to tympanum; limbs with faint brown transverse bands; throat, chest, belly and ventral surface of limbs and tail pale brown or cream, with scattered dark spots on belly and thighs (dark spots absent in QCAZ 8428). In addition, juveniles generally have dark brown transverse bands on dorsum, dark flecks on head, and transverse rows of dark brown blotches on flanks. The neck and sides of head have a bright orange tint in one specimen (QCAZ 12535).

#### Distribution and ecology.

*Enyalioides anisolepis* is known to occur between 724–1742 m on the Amazonian slopes of the Andes in southern Ecuador and northern Peru (Fig. 3). It is known from Provincia Zamora-Chinchipe in extreme southern Ecuador and Región Cajamarca in northern Peru. Most specimens were found sleeping at night (7:00 pm–1:00 am) between 0.2–1.5 m above ground on stems, leaves, and tree roots in primary and secondary forests. Nine of the 15 known specimens were found within 5 m of small streams.

#### Etymology.

The specific epithet *anisolepis* is a noun (in apposition) in the nominative singular and derives from the Greek words *anisos* (= unequal) and *lepis* (= scale). It refers to the heterogeneous scales on the dorsum, flanks and hind limbs of lizards of this species.

### Enyalioides sophiarothschildae sp. n.

Taxon classificationAnimaliaSquamataHoplocercidae

http://zoobank.org/451884BA-28DE-4974-A7D1-FB4F77680FA7

Enyalioides sophiarothschildae Proposed standard English name: Rothschild’s woodlizards
Enyalioides sophiarothschildae Proposed standard Spanish name: lagartijas de palo de Rothschild

#### Type material.

**Figure 8.**
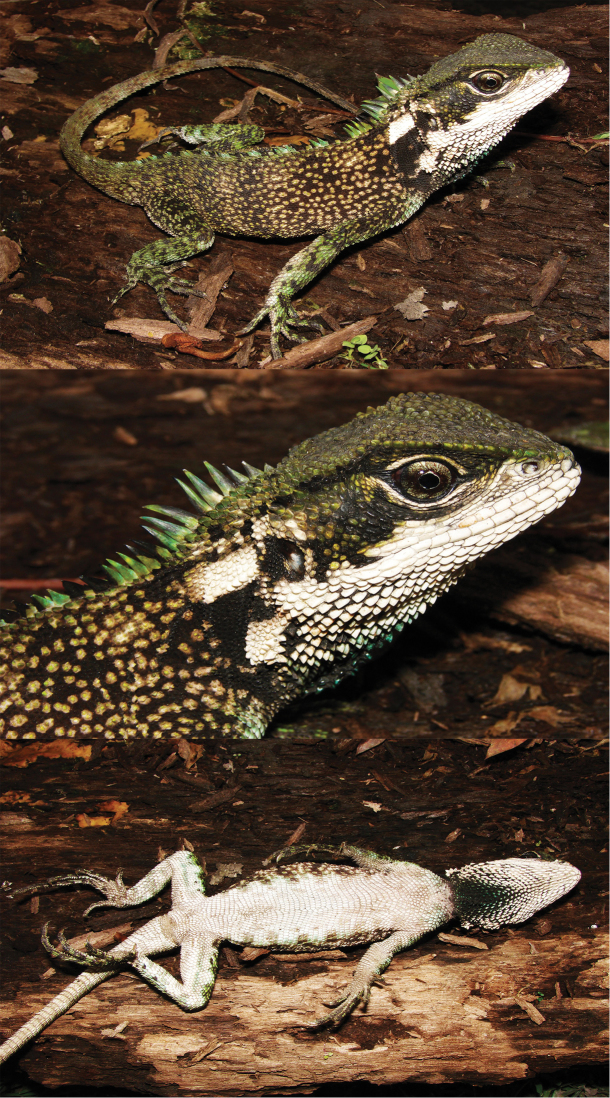
Holotype of *Enyalioides sophiarothschildae* sp. n. (CORBIDI 647, adult male, SVL = 135 mm). Top: dorsolateral view; middle: lateral view of head; bottom: ventral view. Photograph by Pablo J. Venegas.

*Holotype*. CORBIDI 647 (Fig. 8), an adult male from Río Lejía in the trail La Cueva-Añazco Pueblo, -6.83655°S; -77.48603°W (DD), 1700 m, Provincia Mariscal Cáceres, Región San Martín, Perú, collected on 2 February 2008 by P.J. Venegas.

*Paratypes (2)*. PERU: Región San Martín: Provincia Mariscal Cáceres: MUSM 21883-84, adult males, El Dorado, -6.76666°S; -77.54500°W, 1600m, collected on 5 December 2003 by P.J. Venegas.

#### Diagnosis.

*Enyalioides sophiarothschildae* can be distinguished from other species of *Enyalioides*, except for *Enyalioides laticeps*, by having caudal scales that are relatively homogeneous in size on each caudal segment; in all other species of *Enyalioides*, the dorsal and lateral caudals increase in size posteriorly on each caudal segment, and the largest (posteriormost) caudals on each segment are mucronate or have some kind of projection (Torres-Carvajal et al. 2011). *Enyalioides sophiarothschildae* differs from *Enyalioides laticeps* (character states in parentheses) in color patterns: gular region in males white with a black medial patch scattered with turquoise scales (orange or dirty cream with longitudinal brown, reddish-brown, bluish, or orange streaks, and a large brown or black medial blotch at the level of the gular fold); chest in males grayish white with a turquoise tone (usually an orange tone); labials and chin immaculate white (cream or green in many tones, but never immaculate white).

#### Description of holotype.

Male (Fig. 8); SVL = 135 mm; TL = 223 mm; maximum head width = 28.4 mm; head length = 34.7 mm; head height = 24.3 mm; dorsal head scales uni- or multicarinate, projected dorsally; parietal eye present; 3–4 scales immediately posterior to superciliaries conical, dorsolaterally projected, and slightly larger than adjacent scales; temporal scales small, multicarinate, separated from each other by tiny granular scales; no distinctly enlarged pretympanic scales; superciliaries 13; canthals six; postrostrals three; supralabials 10 if counted to a point below middle of eye; rostral (3.14 mm wide × 1.47 mm high) slightly wider than adjacent supralabials; single longitudinal row of lorilabials between suboculars and supralabials at level of middle of eye, two longitudinal rows of lorilabials anterior to this point; loreal region composed of small, smooth, and juxtaposed scales, some of which are separated from each other by tiny granular scales; nasal at level of supralabials III–IV; left and right infralabials nine if counted to a point below middle of eye; mental (2.77 mm wide × 2.60 mm high) slightly wider and 1.5 times higher than adjacent infralabials; postmentals two; gulars ventrally projected and separated from each other by skin covered with tiny granular scales; gular fold complete midventrally, extending dorsally and posteriorly to form antehumeral fold; neck with several oblique folds, and a dorsolateral row of enlarged scales; distal part of oblique fold immediately anterior to antehumeral fold with approximately 10 enlarged scales similar in size to gulars, but more than twice the size of adjacent fold scales.

Vertebral crest strongly projected and decreasing in size posteriorly, with vertebrals on neck at least four times higher than those between hind limbs; crest bifurcates posteriorly and extends onto tail less than ¼ its length; body flanks between fore and hind limbs with slight dorsolateral and ventrolateral longitudinal folds; scales on dorsolateral folds similar in size to adjacent scales; dorsal and flank scales small, keeled, imbricate, more or less homogeneous in size, and separated from each other by skin covered with tiny granular scales; ventral scales imbricate, smooth or slightly keeled, rectangular or rhomboid, with a posterolateral mucron; ventrals more than twice the area of dorsals.

Limb scales keeled dorsally and smooth or slightly keeled ventrally; scales on dorsal and posterior surfaces of thighs heterogeneous in size, with most scales less than half the size of those on anterior and ventral surfaces, separated from each other by skin covered with tiny granular scales; subdigitals on finger IV 17; subdigitals on toe IV 25; femoral pores on each side four; tail laterally compressed and gradually tapering posteriorly; caudal scales strongly keeled and imbricate, not gradually increasing in size posteriorly on lateral and dorsal aspects of each caudal segment; caudals larger ventrally than dorsally; individual caudal segments three scales long ventrally and six scales long dorsally.

#### Color in life of holotype

(Fig. 8). Head dark green with large black blotch between the eye and the tympanum; loreal region, nasal scale, labials and chin white; white blotch on posterior end of mandible; neck greenish brown dorsally and dark brown laterally, with a white rhomboidal blotch extending longitudinally from tympanum to scapular region; dorsal body background dark brown with scattered green scales and pale spots; limbs dark brown with green transverse bands; tail dark green with scattered dark brown marks; vertebral crest with intermixed green and dark brown scales; gular region white with a black posteromedial patch bearing scattered turquoise scales; chest grayish white with a turquoise tone anteriorly; belly grayish white with scattered, faint, pale brown blotches; ventral surface of limbs grayish white, with a longitudinal faint turquoise stripe along the thighs; tail grayish white; iris silver peripherally and dark brown centrally, with dark brown reticulations; silver ring around pupil.

#### Variation.

Variation in meristic and morphometric characters of *Enyalioides sophiarothschildae* are presented in Table 2. One male paratype (MUSM 21883) differs from the holotype in having some scattered dark brown blotches on the throat.

#### Distribution and ecology.

*Enyalioides sophiarothschildae* is known from the northeastern slopes of the Cordillera Central in Peru between 1600–1700 m (Fig. 3). This species is only known from two adjacent localities, the trail to La Cueva-Añasco Pueblo in the drainage of the Lejía river and El Dorado in the drainage of the Blanco river, both tributaries of the Huallabamba river in the northern part of the Huallaga river basin. This area corresponds to the Selva Alta (400–1000 m) and Yungas (300–2300 m) ecoregions (Brack 1986; Peñaherrera del Aguila 1989).

Individuals of *Enyalioides sophiarothschildae* were found active by day in primary forest. The holotype was found crossing a trail and tried to hide between the roots of a big tree when approached for capture. One of the paratypes climbed up a tree three meters above the ground when approached. The other paratype was found sitting on a big root.

#### Etymology.

The specific epithet is a noun in the genitive case and is a patronym honoring Sophia Rothschild in recognition of her financial support for the improvement of the herpetological collection of CORBIDI through the BIOPAT Program.

### Phylogenetic relationships

**Figure 9.**
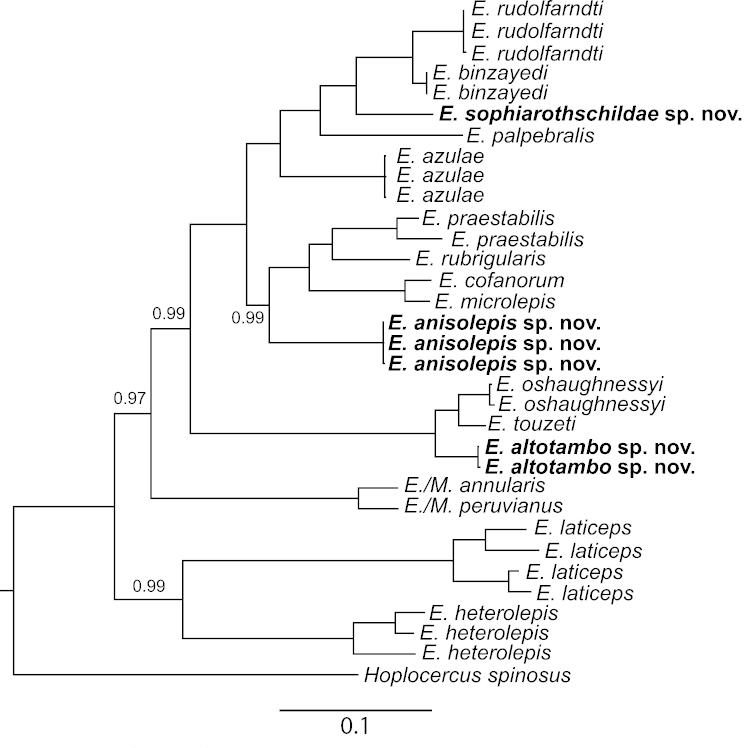
50% Majority rule consensus tree of hoplocercine lizards (*Enyalioides* = *Enyalioides*, *Morunasaurus* = *Morunasaurus*) based on a Bayesian analysis of mtDNA sequences. Posterior probabilities are equal to 1, unless otherwise noted by numbers next to branches. Outgroup taxa are not shown. The notation *Enyalioides/Morunasaurus* indicates that according to the phylogenetic definitions (de Queiroz and Gauthier 1990) of the names *Enyalioides* and *Morunasaurus* proposed by Torres-Carvajal et al. (2011), *Morunasaurus* is a subclade of *Enyalioides*.

The phylogenetic tree inferred in this study (Fig. 9) is consistent with Torres-Carvajal and de Queiroz’s (2009) phylogenetic hypothesis in that species of *Enyalioides* are split into two primary subclades: one containing *Enyalioides heterolepis* and *Enyalioides laticeps* as sister taxa, and the other including all remaining species of *Enyalioides*, as well as possibly *Morunasaurus*. All species described in this paper are nested within the second clade.

Torres-Carvajal and de Queiroz (2009; see their Fig. 5) found *Enyalioides oshaughnessyi* to be paraphyletic relative to *Enyalioides touzeti* based on three samples of *Enyalioides oshaughnessyi* and one sample of *Enyalioides touzeti*. They hypothesized that either *Enyalioides oshaughnessyi* as previously circumscribed represents a single species, but its mtDNA has not yet become monophyletic relative to that of *Enyalioides touzeti*, or *Enyalioides oshaughnessyi* represents more than one species. Torres-Carvajal et al. (2011) found support for the latter hypothesis based on the color of the iris, red in both sexes of most specimens of *Enyalioides oshaughnessyi* and reddish brown in two adult specimens from Alto Tambo (*Enyalioides altotambo* type specimens). Addition of sequence data from a second specimen from Alto Tambo further supports this hypothesis in that *Enyalioides oshaughnessyi* and *Enyalioides altotambo* are reciprocally monophyletic. *Enyalioides altotambo* is strongly supported as monophyletic (PP = 1) and, in agreement with previous phylogenetic hypotheses (Torres-Carvajal and de Queiroz 2009; Venegas et al. 2013), is sister (PP= 1) to a clade (PP = 1) formed by *Enyalioides touzeti* and *Enyalioides oshaughnessyi* (Fig. 9).

*Enyalioides anisolepis* is strongly supported (PP = 1) as monophyletic and is sister (PP = 0.99) to a clade (PP = 1) composed of *Enyalioides cofanorum*, *Enyalioides microlepis*, *Enyalioides rubrigularis*, and *Enyalioides praestabilis*. *Enyalioides sophiarothschildae* is sister (PP = 1) to a clade (PP = 1) composed of two recently discovered species, *Enyalioides binzayedi* and *Enyalioides rudolfarndti* (Venegas et al. 2011; Venegas et al. 2013) from Peru. Thus, the phylogenetic tree presented here strongly supports both referral of the three newly discovered species to *Enyalioides* and their status as different species from those recognized previously. Differences in morphology and color patterns presented above provide additional evidence for recognizing *Enyalioides altotambo*, *Enyalioides anisolepis* and *Enyalioides sophiarothschildae* as species.

### Key to the 19 species of Hoplocercinae

The following key is artificial in the sense that its structure does not necessarily reflect the order of branching in the phylogeny.

**Table:** 

1	Dorsal head scales flat, smooth, juxtaposed; vertebral crest absent or composed of a discontinuous row of enlarged scales that are longer than tall	**2**
–	Dorsal head scales conical; vertebral crest present, composed of projecting scales that are taller than long	**5**
2	Tail depressed, short (tail length < snout-vent length), with enlarged spiny scales dorsally and laterally	***Hoplocercus spinosus***
–	Tail nearly round, moderate (tail length > snout-vent length), with rings of enlarged spiny scales	**3**
3	Vertebral region of trunk without enlarged scales; tail with three scale rows separating the spiny whorls ventrally	***Morunasaurus groi***
–	Some vertebral scales in trunk region enlarged forming a discontinuous longitudinal row; tail with two scale rows separating the spiny whorls ventrally	**4**
4	Usually two femoral pores on each leg; two postmentals; females without streaks on throat	***Morunasaurus annularis***
–	Femoral pores 3–4 on each leg; usually four postmentals; females with dark streaks on throat	***Morunasaurus peruvianus***
5	Caudal scales homogeneous in size within each autotomic segment	**6**
–	Caudal scales increase in size posteriorly within each autotomic segment	**7**
6	Gular region in males white with a black medial patch	***Enyalioides sophiarothschildae***
–	Gular region in males orange or dirty cream, with longitudinal brown, reddish-brown, bluish, or orange streaks, and a large brown or black medial blotch at the level of the gular fold	***Enyalioides laticeps***
7	Lateral superciliary projection present; vertebral crest usually discontinuous (absent on posterior part of neck)	***Enyalioides palpebralis***
–	Lateral superciliary projection absent; vertebral crest continuous	**8**
8	Scattered, conspicuous large scales on dorsum, flanks, and hind limbs present	**9**
–	Scattered, conspicuous large scales on dorsum, flanks, and hind limbs absent	**10**
9	Scattered large scales tetrahedral in shape; vertebrals on neck maximum twice as high as those between hind limbs	***Enyalioides heterolepis***
–	Scattered large scales strongly keeled, not tetrahedral in shape; vertebrals on neck at least three times higher than those between hind limbs	***Enyalioides anisolepis***
10	Ventrals smooth or slightly keeled	**11**
–	Ventrals conspicuously keeled	**12**
11	Gulars in males cream or yellow without black margins; usually one femoral pore on each leg	***Enyalioides praestabilis***
–	Gulars in males bright orange or red, with black margins; usually two femoral pores on each leg	***Enyalioides rubrigularis***
12	Dorsals heterogeneous in size, with scattered, tetrahedral, projecting scales (sometimes absent in males or juveniles); dorsolateral crests well developed between hind limbs	***Enyalioides cofanorum***
–	Dorsals homogeneous in size, without projecting scales; dorsolateral crests inconspicuous or absent between hind limbs	**13**
13	Dorsals smooth or slightly keeled	**14**
–	Dorsals conspicuously keeled	**15**
14	Scales on flanks heterogeneous in size, with a few enlarged, circular, keeled scales; iris bright red in both sexes; black patch under gular fold extending dorsally to form a short antehumeral bar in males	***Enyalioides oshaughnessyi***
–	Scales on flanks almost homogenous in size; iris brown in both sexes; black medial patch on gular region not extending dorsally to form an antehumeral bar in males	***Enyalioides altotambo***
15	Dorsals in transverse row between dorsolateral crests at midbody 31 or fewer	**16**
–	Dorsals in transverse row between dorsolateral crests at midbody more than 31	**17**
16	Scales along the lateral edge of the skull roof strongly projected; dorsal scales homogeneous in size, with prominent median keel; antehumeral orange blotch in adult males absent	***Enyalioides binzayedi***
–	Scales along the lateral edge of the skull roof slightly projected; dorsal scales heterogeneous in size, without prominent median keel; distinct antehumeral orange blotch in adult males	***Enyalioides rudolfarndti***
17	White or cream spot posterior to tympanum usually present; 41–54 (mean = 45.96 ± 3.49) dorsals in transverse row between dorsolateral crests at midbody; gular background in adult males light blue	***Enyalioides microlepis***
–	White or cream spot posterior to tympanum absent; 37–47 (means = 41.63 ± 3.20 in *Enyalioides azulae*, 40.50 ± 1.90 in *Enyalioides touzeti*) dorsals in transverse row between dorsolateral crests at midbody; gular background in adult males cream or black	**18**
18	Vertebral scales in neck region in adult males similar in size to vertebrals in pelvic region; 45–57 (mean = 51.13 ± 4.05) gulars	***Enyalioides azulae***
–	Vertebral scales in neck region in adult males more than twice as high as vertebrals in pelvic region; 42–48 (mean = 44.40 ± 2.22) gulars	***Enyalioides touzeti***

## Supplementary Material

XML Treatment for Enyalioides altotambo

XML Treatment for Enyalioides anisolepis

XML Treatment for Enyalioides sophiarothschildae

## Appendix

**Specimens examined**

*Enyalioides cofanorum*.—**COLOMBIA:** *Amazonas*: Puerto Nariño, 3°46'13"S, 70°22'59"W, 110 m, ICN 4229; **ECUADOR:** *Orellana*: 4km N Anangu on Garza Cocha at Hosteria La Selva, 260 m, MCZ 174428–29; Cononaco, QCAZ 5975; SPF, QCAZ 2710; transecto PBT, Pozo Capiron 2, QCAZ 7563; Tiputini Biodiversity Station, 0°37'5"S, 76°10'19"W, 215 m, QCAZ 8006; Yasuni National Park, bloque Shiripuno, 0°43'35"S, 76°43'36"W, QCAZ 3521; *Sucumbíos*: La Selva lodge, 0°24'0"S, 76°39'0"W, QCAZ 2935, 2961, 3951, 3953; Limoncocha, MCZ 157697; Santa Cecilia, 0°5'6"N, 76°59'33"W, 340 m, KU 105342 [paratype], 112180–81 [paratypes], 122118 [paratype], 146658 [holotype], 147584–85 [paratypes], 175308; Tarapoa, 0°7'60"S, 76°25'0"W, 283 m, FHGO 5764; *Zamora Chinchipe*: cuenca del rio Jamboe, Sakantza, 1230 m, FHGO 2342; **PERU:** *Loreto*: Ampiyacu river, Distrito Pevas, 3°19'0"S, 71°51'0"W, 100 m, CAS 8323.

*Enyalioides heterolepis*.—**COLOMBIA:** *Antioquia*: Dabeiba, río Amparradó, campamento Ingeominas, 6°42'0"N, 76°27'0"W, 805 m, ICN unassigned numbers (2 specimens); Municipio Frontino, corregimiento La Blanquita (Murrí), 800 m, IND-R 4229; Municipio Frontino, Vereda Venados, Parque Nacional Natural Las Orquídeas, afluente de la quebrada El Retiro, 6°33'0"N, 76°18'25"W, 850–950 m, ICN 9143; *Cauca*: bajo Calima, granja de la Secretaría de Fomento, 3°59'47"N, 76°58'28"W, ICN 4231; Guapí, 2°33'23"N, 77°51'50"W, ICN 4232, 4234–35; Gorgona Island, 2°58'31"N, 78°12'27"W, 30–120 m, FMNH 165387–88, ICN 824, 826–27, 832–38, 1045, 1247–53, 1325–26, 4237–44, 4521, 6515, ICN unassigned numbers (2 specimens), KU 192676–77; Guapí, on road to pipeline between Chansará-Cantadelicia, ICN 4233, 4236; Municipio de Junta, headwaters río Guapi, IND-R 3570; Quebrada Guangui, ICN unassigned number (1 specimen); *Chocó*: 5 km NW Playa de Oro, IND-R 3556; Bahía Solano, Parque Nacional Natural Utría, ICN unassigned number (1 specimen); headwaters of río San Juan, ca. 800 m, FMNH 165224; Quibdo, San josé de Purré, río Cabi, IND-R 5035; Serranía del Baudo, Alto del Buey, 6°6'0"N, 77°13'0"W, ICN 4245–46; *Valle*: Virology Field Station, USNM 151610–12; *Valle del Cauca*: 8 km W Danubio, río Anchicaya, KU 169853; Buenaventura, Base Naval Málaga, quebrada Valencia, 3°58'0"N, 77°18'0"W, ICN unassigned numbers (2 specimens); Dagua, Vereda La Elsa, 3°34'47"N, 76°46'54"W, 980 m, ICN 9091; km 6 on road Buenaventura-río Calima, 3°53'36"N, 77°4'11"W, 0 m, FMNH 165181–82; km 22 on road Buenaventura-río Calima, FMNH 165223; Municipio Restrepo, Vereda Alegre, Campo Chanco, 3°38'14"N, 76°13'44"W, 460 m, ICN 9093; río Raposo, above caserío El Tigre, 3°42'0"N, 77°5'60"W, 11 m, ICN 1501–02; *no specific locality*: ICN 9092, 9801, 11313; **ECUADOR:** *El Oro*: Gualtaco, USNM 211076; *Esmeraldas*: Alto Tambo, 253 m, QCAZ 5523; Bosque Protector La Chiquita, 30 km E San Lorenzo on road to Ibarra, QCAZ 3839; Corriente Grande, 70 m, QCAZ 3531; Jatun Sacha Field Station, Montañas Mache-Chindul, 41km W Quinindé, 0°21'21"N, 79°42'12"W, 600 m, FHGO 3200; Loma Linda, río Onzole, 95 m, QCAZ 3626; Mayronga, 100 m, QCAZ 2185–86, 2263–66; Reserva Ecológica Mache-Chindul, comunidad San Salvador, FHGO 4063; San Miguel de Cayapas, QCAZ 412; *Los Ríos*: Estación Biológica Río Palenque, 150-220 m, KU 146657, 164166, 180657–58, QCAZ 427, USNM 285451, 285454; *Manabí*: 38 km NW El Carmen, ca. El Carmen-Pedernales road, 330 m, KU 218380; *Pichincha*: 15 km NW La Florida, QCAZ 2844; La Perla, QCAZ 2025–26; Palma Real, USNM 211094; Puerto Quito, km 132 on road Calacalí-La Independencia, Hostería Selva Virgen, FHGO 4314; río Blanco, below mouth of río Toachi, USNM 211088; río Caoni, USNM 211095; río Toachi, between kms 100-110 on road to Santo Domingo de Los Colorados, USNM 211097–98; *Santo Domingo de los Tsáchilas*: km 30 Quinindé-Santo Domingo de los Colorados, USNM 211091; Santo Domingo de los Colorados, 600 m, KU 121090–91; PANAMA: *Colón*: Achiote, 40 m, KU 96688; *Darién*: Laguna, 820 m, KU 76050–53; ridge btw río Jaque & río Imamado, 730 m, KU 113490–94; SE slope Cerro Pirre, 1060 m, KU 96689–90; Tacarcuna, 550 m, KU 76047–48; *San Blas*: Armila, USNM 150121 *Veragua* (possibly Veraguas): MHNP 4067 [holotype].

*Enyalioides laticeps*.—**BRAZIL:** Fonteboa, upper Amazon, MHNP 6821 [holotype]; **COLOMBIA:** *Amazonas*: 50 km N Chorrera on Igará-Paraná, IND-R 1038–41; headwaters of río Caiwima, tributary of río Amacayacu, ca. 70 km NNE Puerto Nariño, MCZ 154482; Leticia, 4°12'55"S, 69°56'26"W, 83 m, ICN unassigned number (1 specimen); Parque Nacional Natural Amacayacu, cabaña Amacayacu, IND-R 4195; Parque Nacional Natural Amacayacu, río Amacayacu, Puerto Mogue, close to río Cabimas, IND-R 1037; Parque Nacional Natural Amacayacu, Mata-mata creek, 3 km W Mata-mata cabin, 3°41'0"S, 70°15'0"W, 150 m, ICN 9094; Puerto Rastrojo, río Miriti, IND-R 1920, 1929; río Amacayacu, tributary of río Amazonas, ca. 50km NNE Puerto Nariño, MCZ 156348; río Amacayacu-Caiwima, ca. 40km NNE Puerto Nariño, MCZ 154481; río Miriti Paraná, IND-R 1905; *Caquetá*: 30 km from mouth of río Cuemani, IND-R 1063–65; Florencia, MLS 117; Parque Nacional Natural Chiribiquete, río Mesay, Puerto Abeja, 0°5'27"N, 72°25'0"W, IAvH 4746; *Guaviare*: Chiribiquete, ICN unassigned number (1 specimen); *Meta*: río Guayabero, Angostura No.1, 2°17'0"N, 73°58'0"W, 300–350 m, ICN 1270; Cumaral, Vereda Juan Pablo II, 3°47'0"N, 73°55'0"W, ICN 7255; Guaguriba on road to Acacias, MCZ 156323; La Macarena, campamento Isama, río Duda, ICN 4230; La Macarena, Caño Guapayita, ICN 677; Las Salinas, 3 km NW Restrepo, 4°16'9"N, 73°35'9"W, 720 m, ICN unassigned number (1 specimen); La Macarena, río Duda, Parque Nacional Natural Los Tiniguas, campamento de primatología Puerto Chamuza, IND-R 4019–22, 4034; Serranía La Macarena, Caño Sardinata, 30 km W Vista Hermosa, IND-R 287; Villavicencio, 4°9'12"N, 73°38'6"W, 500 m, AMNH 35277, FMNH 30815, MLS 116; Villavicencio, Pozo Azul creek, 4°9'12"N, 73°38'6"W, 500 m, ICN 8341, ICN unsassigned numbers (2 specimens), MCZ 154334; *Putumayo*: ca. 10 km (airline) S Mocoa, 700–800 m, AMNH 106631; no specific locality: FMNH 165208, 165211; *Vaupés*: Caparú, surroundings of lake Taraira, 1°8'46"S, 69°29'14"W, ICN 8058–61; Estación Biológica Caparú, IND-R 4382; **ECUADOR:** *Morona Santiago*: cantón Taisha, parroquia Macuma, centro Shuar Macuma, 2°8'6.6"S, 77°42'54"W, 720 m, FHGO 5460; Arapicos, 1°51'0"S, 77°57'0"W, 981 m, USNM 211111; *Napo*: Ahuano, QCAZ 7014; Ávila, río Napo, CAS-SUR 8261–62; Tena, QCAZ 6054; *Orellana*: 7 km S río Tiputini, KU 299832–33; Estación Científica Yasuní, QCAZ 7388; Loreto, 0°40'0"S, 77°19'0"W, 451 m, USNM 211121; Parque Nacional Yasuni, Tambococha, FHGO 3692; Parque Nacional Yasuni, Tiputini, Ishpingo, FHGO 5346; Río Napo, Añangu, south bank, QCAZ 9503; Sacha Lodge, QCAZ 8884; *Pastaza*: Alto río, USNM 211146; Lorocachi, QCAZ 3222; Palmira, río Pastaza valley, AMNH 37554; río Capahuari, USNM 211122; río Huiyayacu, USNM 211128; río Pindo, USNM 211143; río Shiripuno, 1°5'0"S, 76°50'0"W, FHGO 1624; Sarayacu, USNM 211124; Villano, 1°30'0"S, 77°29'0"W, 388 m, QCAZ 8118, 8262; *Sucumbíos*: 2 km W Lago Agrio, KU 299835; Lago Agrio, KU 299834, KU 299836; río Cuyabeno, USNM 211113; Santa Cecilia, 0°3'0’’, N, 76°59'0’’, 340 m, KU 122104–05, 122110–11, 147931, 147939–41, 152497; San Jose, S Tarapoa, FHGO 4839; San Pablo de Kantesiaya, 0°15'0"S, 76°25'30"W, 240 m, FHGO 850; Zancudococha, 0°25'0"S, 75°30'0"W, 220 m, FHGO 304; **PERU:** *Amazonas*: Caterpiza, USNM 568575; Galilea, USNM 568576–80; *Cusco*: Pagoreni, río Camisea, 11°42'23"S, 72°54'11"W, 465m; *Loreto*: Explorama Lodge, jct río Yanamono & río Amazonas, KU 220493; Intuto, río Tigre, AMNH 60575; San Jacinto, 175 m, KU 222164; *San Martin*: San Martin, 14 km ESE Shapaja, 360 m, KU 212627; *Ucayali*: río Calleria, Colonia Calleria, 15 km from Ucayali, CAS 95143. NO SPECIFIC LOCALITY: ICN 1231.

*Enyalioides oshaughnessyi*.—**ECUADOR:** *Esmeraldas*: Bilsa Ecological Reserve, 225 m, QCAZ 6866; *Guayas*: cerro Masvale, QCAZ 9893; *Los Ríos*: Estacion Biológica Río Palenque, 150–220 m, KU 152597, USNM 285456–57, Estación Biológica Jauneche, 50 m, QCAZ 6899; *Pichincha*: Finca Victoria, 37 km SE Santo Domingo de los Colorados, MCZ 80958; Hotel Tinalandia, 15 km SE Santo Domingo de los Colorados, MCZ 145269; Puerto Quito, MCZ 164509; Recinto Playa Rica, on road Nanegal-Selva Alegre, QCAZ 7426; Silanchi, río Blanco, USNM 211102; Tandapi, MCZ 164789; Unión del Toachi, 300 m, QCAZ 5326, 6682; *Santo Domingo de los Tsáchilas*: 1 km N, 2 km E Santo Domingo de los Colorados, 620 m, KU 179417; 2 km E, 1 km S Santo Domingo de los Colorados, 600 m, KU 179416; Finca La Esperanza, 5 km W Santo Domingo de los Colorados, USNM 211105; Finca La Esperanza, 5 km W Santo Domingo de los Colorados, USNM 211106–07; Santo Domingo de los Colorados, KU 109630, USNM 211103, 211109. NO SPECIFIC LOCALITY: USNM 22448, 22450.
